# Anticancer and Antimicrobial Properties of Inorganic Compounds/Nanomaterials

**DOI:** 10.1155/2019/6019632

**Published:** 2019-06-09

**Authors:** Rais Ahmad Khan, Aurel Tăbăcaru, Farman Ali, Bon H. Koo

**Affiliations:** ^1^Department of Chemistry, College of Science, King Saud University, Riyadh 11451, Saudi Arabia; ^2^Department of Chemistry, Physics, and Environment, Faculty of Sciences and Environment, “Dunarea de Jos” University of Galati, 111 Domneasca Street, Galati 800201, Romania; ^3^Department of Applied Chemistry, Aligarh Muslim University, Aligarh 202001, India; ^4^School of Materials Science and Engineering, Changwon National University, Changwon 51140, Republic of Korea

The field of bioinorganic chemistry has emerged after a serendipitous discovery of cisplatin by Rosenberg in 1962. Cisplatin is called “penicillin of cancer” because of its wide application and was a first chemotherapeutic drug. After this, a new field of drug formulation with metal center/core was sparked and investigated. Interestingly, it became the promising alternative for the treatment of various diseases like cancer, microbial, quorum sensing, and biofilm-associated infectious disease. The field of metallodrugs has also evolved with the emergence of nanomaterials as therapeutic anticancer and antibacterial agents. These molecules have also shown significant potential to act as potential drug candidates in different prospects from chemotherapy to diagnostics to drug delivery in various diseases. Nanomaterials possess unique physical and chemical properties. The fabrication of nanomaterials gives rise to controlled size, shape, compositions, charge, aggregation, and solubility. This special issue includes a collection of original papers covering aspects related to the synthesis and characterization of various metal-based compounds and nanomaterials with anticancer and antimicrobial properties.

The critical review by N. Hlapisi et al. explores the utilization of gold nanorods, as delivery and photothermal agents, and porphyrins, as photosensitizers, in the treatment of cancer and bacterial diseases, delving into ways of incorporating both photothermal and photodynamic therapies with the scope of producing less toxic, more efficient, and specific compounds for the treatment. The excellent imaging and sensing properties shown by gold nanorods qualify them as excellent for photothermal therapy. On the other hand, porphyrin and nanorod conjugates show excellent properties as photosensitizers due to the fact that they have more than one absorption band on the near infrared region; hence, they can be manipulated and are used to penetrate deeper into tissue for photodynamic therapy.

The new inorganic-organic hybrid compound, [{Cu(phen)_2_}_2_(H_4_W_12_O_40_)], was synthesized and structurally characterized by D. Li et al. The Keggin anion H_4_W_12_O_40_^4−^ from the constitution of this compound was grafted with two coordination units {Cu(phen)_2_}, forming an electrically neutral molecule. The antibacterial activity of several polyoxometalate compounds with different anionic structures, also including the new compound, was studied. The results showed that the new polyoxometalate-based compound (POM) could inhibit the growth of *Enterococcus faecalis* FA2 strains and that the antibacterial activity of the polyoxometalate compounds is dependent on the component elements of POM but is less relative with the anion structures.

In the study of N. N. Farshori et al., silver nanoparticles (ND-AgNPs) were synthesized using an aqueous extract of *Nepeta deflersiana* plant, and their morpho-structural features were revealed by ultraviolet-visible spectroscopy, Fourier transforms infrared spectroscopy, X-ray diffraction, transmission electron microscopy, scanning electron microscopy, and energy dispersive spectroscopy. The results obtained from these various characterization tools showed that the average size of synthesized AgNPs was 33 nm and in face-centered-cubic structure. The anticancer potential of ND-AgNPs was investigated against human cervical cancer cells (HeLa). The cytotoxic response was assessed by 3-(4,5-dimethylthiazol-2-yl)-2,5-diphenyltetrazolium bromide (MTT), neutral red uptake (NRU) assays, and morphological changes. The influence of cytotoxic concentrations of ND-AgNPs on oxidative stress markers, reactive oxygen species (ROS) generation, mitochondrial membrane potential (MMP), cell cycle arrest, and apoptosis/necrosis was also studied. The cytotoxic response observed was in a concentration-dependent manner. Furthermore, the results also showed a significant increase in ROS and lipid peroxidation (LPO), along with a decrease in MMP and glutathione (GSH) levels. The cell cycle analysis and apoptosis/necrosis assay data exhibited ND-AgNPs-induced SubG1 arrest and apoptotic/necrotic cell death. The biosynthesized AgNPs-induced cell death in HeLa cells suggested the anticancer potential of ND-AgNPs, thus suggesting their potential use in the treatment of cervical cancer cells.

Another green method to obtain silver nanoparticles, with cytotoxic and bactericidal properties, was reported by F. Ruiz et al., who used the *Annona muricata* aqueous extract, along with the functionalization with 5-fluorouracil (5-FU). The processes of reduction, nucleation, and functionalization of the obtained AgNPs were analyzed by UV-Vis absorption spectroscopy, and it was found that they are a function of the contact time of the metal ions with the extract. The morpho-structural characterization of AgNPs carried out by transmission electron microscopy revealed their quasispherical shape with an average particle size of 10.87 nm. The recorded *Z*-potential had a value of −27.3 ± 1.22 mV, demonstrating repulsion between the particles and good colloidal stability of the nanomaterials. The antibacterial properties of the synthesized nanomaterials showed significant inhibition against *Enterococcus faecalis*, *Staphylococcus aureus*, and *Escherichia coli*. The cytotoxicity of the AgNPs at 24 and 48 hrs displayed an increment in cell viability associated with the particle functionalization by 5-FU, and only a few dead cells at 24 hrs were observed in the fluorescence microscopy images.

The research article of T. Perera et al. focuses on the synthesis, characterization, and antimicrobial activity of four novel water-soluble copper-triazine derivatives in search of potent antibacterial and antifungal drug leads. As such, 3-(2-pyridyl)-5,6-diphenyl-1,2,4-triazine-4,4′-disulfonic acid monosodium salt (*L*1, ferrozine) and 3-(2-pyridyl)-5,6-di(2-furyl)-1,2,4-triazine-5,5′-disulfonic acid disodium salt (*L*2, ferene) have been used as ligands to study the complexation towards copper(II). The synthesized complexes, [CuCl_2_(ferrozine)]·7H_2_O·MeOH (1), [CuCl_2_(ferrozine)_2_]·5H_2_O·MeOH (2), [CuCl_2_(ferene)]·H_2_O·MeOH (3), and [CuCl_2_(ferene)_2_]·H_2_O·MeOH (4), have been characterized spectroscopically, and preliminary bioassays have been carried out. Complexes (1) and (2) have shown antibacterial activity for both *Staphylococcus aureus* and *Escherichia coli* at 1 mg/disc concentration, and ferrozine has shown a larger inhibition zone against the clinical sample of *Candida albicans* at 1 mg/disc concentration in comparison with the positive control, fluconazole.

This study of Q. Li et al. analyzed the interaction between the novel patent diorganotin(IV) compound bis-[2,6-difluoro-N-(hydroxyl-<*κ*>O)benzamidato-<*κ*>O] (DBDF2,6T) and the hPPARγ protein under physiological condition with the methods of fluorescence quenching, 3D fluorescence, DARTS, ultrafiltration-LC, and computer molecular docking. According to the experimental spectroscopic data, DBDF2,6T could interact with the hPPAR*γ* protein and formed a nonradiative ground-state complex of hPPAR*γ*-DBDF2,6T, mainly through hydrophobic force. The experiments of DARTS and ultrafiltration-LC preliminarily proved the possibility of DBDF2,6T to be an agonist compound to hPPAR*γ* protein. Considering the anticancer activity of DBDF2,6T and various physiological functions performed by agonists of PPAR*γ* protein, the authors concluded that DBDF2,6T had a possibility to interact with the hPPAR*γ* protein as an agonist and finally inducing physiological effects such as anticancer activity.

The novel imidazole salts 1,3-bis(2-hydroxyethyl) imidazolidinium bromide (*L*_A_), 3-(2-ethoxy-2-oxoethly)-1-(3-aminopropyl)-1H-imidazol-3-ium bromide (*L*_B_), 1,3-bis(2-carboxyethyl)-4-methyl-1H-imidazol-3-ium bromide (*L*_C_), and 3-(2-carboxyethyl)-1-(3-aminopropyl)-1H-imidazol-3-ium bromide (*L*_D_), along with their synthesis and characterization, were reported by G. Uluçam and M. Turkyilmaz. The antimicrobial and cytotoxic activities of the synthesized salts on some specific bacteria and cancer cell lines were measured using spectrophotometric methods, showing that *L*_A_ exhibited better inhibition than the selected antibiotic on *Bacillus cereus*, while it is active on the selected bacteria and the yeast together with *L*_B_. On the cytotoxicity evaluation, *L*_C_ showed considerable inhibition effect on HeLa, as *L*_D_ manifested on Hep G2. Although their IC_50_ doses are quite high in comparison with the similar chemicals in the literature, the cytotoxicity of *L*_C_ and *L*_D_ is affirmed by not causing harmful effect on the healthy MEF cells as much as they do on the cancel cell lines.

We hope that this special issue would shed light on the use of metal-based compounds and nanomaterials as potential candidates for anticancer and antimicrobial properties and attract the attention of the scientific community to further challenges and investigations in this field.

